# High frequency and duration of social media exposure during the COVID- 19 pandemic are associated with mental health problems among employees at an Egyptian university

**DOI:** 10.1186/s42506-025-00191-1

**Published:** 2025-04-29

**Authors:** Ghada O. Wassif, Mohamed Y. El-Awady, Mariam W. Nagi, Isis M. Mossad

**Affiliations:** 1 Community, Environmental and Occupational Medicine Department, Faculty of Medicine, Ain Shams University, Cairo, Egypt; 2 Faculty of Medicine and Health Sciences, Aden University, Aden, Yemen

**Keywords:** COVID- 19 misinformation, Social media use, Mental health, Anxiety, Depression

## Abstract

**Background:**

The COVID-19 pandemic posed a significant threat to public health, particularly mental well-being. Due to the rapid spread of the virus and quarantine restrictions, social media usage increased dramatically. Excessive and unregulated social media use may negatively impact mental health, contributing to heightened feelings of helplessness and anxiety. This study aimed to examine the relationship between the frequency and duration of social media use and symptoms of depression and anxiety among employees at Ain Shams University during the COVID-19 pandemic.

**Methods:**

A cross-sectional analytical study was conducted over 6 months, from September 1, 2021, to March 31, 2022**,** among 405 employees at Ain Shams University. A stratified random sampling method was employed in two stages. First, the university’s faculties were categorized into four disciplines: humanities and social sciences, natural sciences, mathematics, statistics, computer science and engineering, and medical and health sciences. Second, two faculties were randomly selected from each discipline to ensure representative sampling. Data were collected using a structured questionnaire, which included the Generalized Anxiety Disorder-7 (GAD-7) and the Patient Health Questionnaire-9 (PHQ-9) scales to assess anxiety and depression levels.

**Results:**

A total of 405 participants were enrolled, comprising 203 males and 202 females, with a mean age of 42.15 ± 10.72 years (range: 20.00–59.00). The majority (*77%*) held a university degree, and 68.9% were married. A history of COVID-19 infection was reported by 38.3% of participants, and 78.8% used social media to stay updated about the pandemic. Approximately, half of the participants accessed social media three to four times per day**,** spending an average of 2 to 3 h daily. According to the PHQ-9 scale, 40.7% of employees who accessed social media six or more times per day experienced severe depression symptoms. Similarly, based on the GAD-7 scale, 41.6% of employees who accessed social media six or more times daily reported severe anxiety symptoms**.**

**Conclusion:**

The findings suggest a concerning association between frequent COVID-19-related social media exposure and symptoms of depression and anxiety. These results highlight the potential mental health risks associated with excessive social media use, emphasizing the need for mental health awareness programs and strategies to combat misinformation during crises.

## Introduction

Coronavirus (COVID- 19) was first identified as a viral outbreak in Wuhan, China, in December 2019. The number of cases escalated rapidly, and by March 11, the outbreak had spread globally, prompting the WHO to declare COVID- 19 a pandemic affecting 114 nations [1]. Egypt officially reported its first case on February 14, 2020, and by early April, the country had more than 800 confirmed cases and over 50 fatalities [2]. By May 3, 2020, the number of confirmed cases had surged to 6465, with approximately 430 recorded deaths, demonstrating a dramatic increase within a single month [3].

The pandemic significantly impacted people in multiple ways, including their physical and mental health, as well as their financial stability. One of the primary concerns was the pandemic’s effect on mental health, particularly the rise in depression, anxiety, stress, and emotional and behavioral changes [4, 5]. The enforcement of lockdowns and social isolation measures aimed at controlling the virus’s spread further intensified psychological distress [6]. In response to these psychological challenges, many individuals turned to social media platforms such as Facebook, Instagram, and Twitter as a coping mechanism to maintain connections with friends and family [7].

A meta-analysis of 14 cross-sectional studies found a significant association between excessive social media use and deteriorating mental health, highlighting its role in exacerbating psychological distress during the pandemic [8]. In Wuhan, China, the prevalence of depression, anxiety, and combined depression and anxiety disorders (CDA) during the COVID- 19 pandemic was reported at 48.3%, 22.6%, and 19.4%, respectively, with more than 80% of participants reporting regular social media use [9].

A survey conducted among 2032 adult Egyptians revealed that 92.1% of participants used Facebook to stay informed about the COVID- 19 outbreak. Additionally, more than 50% of participants agreed that exposure to pandemic-related news on social media was associated with high levels of anxiety and fear [10]. Similarly, a study in Palestine involving 1067 students aged 6 to 18 found that social media significantly contributed to the spread of COVID- 19-related fear, potentially affecting their psychological and mental health [11]. Another study conducted in Kurdistan, Iraq, indicated that most participants used Facebook to stay updated on the pandemic, with a statistically significant positive correlation (*r* = 0.870) between social media use and the spread of COVID- 19 panic [12].

Despite the growing body of research on social media use and mental health during the pandemic, there remains a scarcity of studies addressing the relationship between the frequency and duration of social media use, depression, and anxiety among employees in the Eastern Mediterranean region. Employees represent a crucial demographic segment to study, as workplace stress, digital connectivity, and job demands may exacerbate the psychological impact of excessive social media exposure. Conducting the present study can inform policy development in organizations, enhance employee well-being and productivity, and provide insights into necessary mental health support systems.

The aim of this study is to examine the relationship between the frequency and duration of social media use and depression and anxiety levels among employees at Ain Shams University during the COVID- 19 pandemic.

## Methods

### Study design and sampling

The present study is a cross-sectional analytical study conducted among administrative employees across various faculties at Ain Shams University. A stratified random sampling method was employed in two stages. First, the university’s 21 faculties were grouped into 4 disciplines: (1) humanities and social sciences, (2) natural sciences, (3) mathematics, statistics, computer science, and engineering, and (4) medical and health sciences. Two faculties were randomly selected from each discipline to ensure a representative sample.

The sample size was calculated using PASS 11.0 (Power Analysis and Sample Size Software, NCSS, LLC, Kaysville, UT, USA) and based on a study carried out by *Bendau *et al*. (2020)* [6] who mentioned that the duration of media usage had a significant positive correlation with the depression score as measured by Patient Health Questionnaire- 9 (PHQ- 9) (*r* = 0.19; *p* < 0.001) and anxiety score as measured by Generalized Anxiety Disorder- 7 (GAD- 7) (*r* = 0.20; *p* < 0.001). A sample size of *405* administrative employees achieves 90% power to detect a difference of − 0.19000 between the null hypothesis correlation of 0.00000 and the alternative hypothesis correlation of 0.19000 using a two-sided hypothesis test with a significance level of 0.01000.

### Study tools

A standardized, self-administered questionnaires were used to manually collect data from participants. The questionnaire consisted of three sections: *Part I*: Sociodemographic information, including age, gender, education level, marital status, history of chronic disease, and history of COVID- 19 infection, *Part II*: Frequency and duration of social media use, and *Part III*: Assessment of anxiety and depression levels using the Generalized Anxiety Disorder- 7 (GAD- 7) and Patient Health Questionnaire- 9 (PHQ- 9) scales, respectively. The Generalized Anxiety Disorder- 7 (GAD- 7) and Patient Health Questionnaire- 9 (PHQ- 9) are widely used, validated tools for assessing anxiety and depression, respectively [13][14]. Both instruments have been translated into Arabic and validated in various populations. For example, studies involving Lebanese psychiatric outpatients and Arabic-speaking cancer patients have confirmed the reliability and validity of the Arabic versions in detecting psychological distress [15][16]. These findings support the suitability of the Arabic GAD- 7 and PHQ- 9 for assessing anxiety and depression in diverse clinical and research settings. Both scales use a 4-point Likert scoring system: 0 = “not at all,” 1 = “several days,” 2 = “more than half the days,” and 3 = “almost every day.” The GAD- 7 scale ranges from 0 to 21, with scores classified as follows: 0–4: “normal,” 5–9: “mild anxiety,” 10–14: “moderate anxiety,” and 15–21: “severe anxiety.” A threshold score of 10 has been shown to provide a sensitivity of 89% and specificity of 82% for detecting anxiety disorders [13, 17]. The PHQ- 9 scale ranges from 0 to 27, with depression scores categorized as follows: 0–4: “normal,” 5–9: “mild depression,” 10–14: “moderate depression,” and 15–27: “severe depression” [14].

### Statistical analysis

The collected data were reviewed, coded, tabulated, and entered into a personal computer for analysis using IBM SPSS version 26.0. The analysis consisted of *descriptive statistics*:

Continuous variables were summarized using the mean, standard deviation (SD), and range. The chi-square test was used to examine associations between qualitative variables. When more than 20% of expected cell counts were less than 5, the Fisher’s exact test was applied. To identify independent predictors of depression and generalized anxiety disorder scores (continuous dependent variable), multiple linear regression analysis was conducted. The independent variables included the following: Age, sex, history of chronic disease, and duration of social media use. To ensure the validity of the multiple linear regression analysis, multicollinearity was assessed using the variance inflation factor (VIF). Variables with VIF values exceeding 10 were considered highly collinear. Marital status and education level showed strong collinearity with age categories and were removed. Similarly, frequency and duration of social media use were highly correlated, so frequency was excluded in favor of duration. After these adjustments, all VIF values were within acceptable limits, improving model accuracy and ensuring reliable estimation of predictors for Generalized Anxiety Disorder (GAD- 7) and depression (PHQ- 9) scores. The level of significance was determined using *p*-value ≤ 0.05.

## Results

The characteristics of the studied employees at Ain Shams University indicate that more than one-fourth (28.1%) were in the age groups of 31–40 and 41–50 years. The sample comprised nearly equal proportions of males (50.1%) and females (49.9%). More than three-fourths (77%) of the participants were university graduates, and over two-thirds (68.9%) were married. Additionally, more than one-third (35.6%) had chronic diseases. Over three-fourths (77.5%) of the participants reported knowing a COVID- 19-positive individual, and more than one-third (38.3%) had previously contracted COVID- 19 (Table 1).

**Table 1:** Characteristics of the studied employees at Ain Shams University, Cairo, Egypt, 2021–2022 (*n* = 405)

Variables	No.	%
**Age (years)** Mean ± SD (42.15 ± 10.72) Range (20.00–59.00)		
20–30	67	16.6
31–40	114	28.1
41–50	114	28.1
≥ 51	110	27.2
**Sex**		
Male	203	50.1
Female	202	49.9
**Education**		
High school	56	13.8
University	312	77.1
Postgraduate studies	37	9.1
**Marital status**		
Single	90	22.2
Married	279	68.9
Divorced/widowed	36	8.9
**Chronic disease**		
No	261	64.4
Yes	144	35.6
**Know a positive Covid- 19 patient**		
No	91	22.5
Yes	314	77.5
**Previously suffered from Covid- 19**		
No	250	61.7
Yes	155	38.3

Regarding the various sources of information, as well as the frequency and duration of social media use among the studied employees, social media platforms such as Facebook and WhatsApp were the most frequently used sources of information (78.8%), followed by official websites (56.0%) and TV programs (50.4%). In contrast, radio programs (8.9%) and newspapers (6.4%) were the least utilized sources.

Concerning social media usage, nearly three-fourths (74.1%) of employees accessed social media at least 3 times per day, with 44.9% using it 3–5 times daily and 29.2% more than 6 times daily. In terms of duration, 47.7% spent 2–3 h per day on social media, while 23.2% exceeded 4 h daily, indicating a high level of engagement with digital platforms for pandemic-related updates (Table 2).

**Table 2:** Sources of information, frequency, and duration of use of social media during Covid- 19 pandemic among studied employees at Ain Shams University (*n* = 405)

Variables	No.	%
**Sources of information#**		
Official websites	227	56.0
Social media like Facebook or WhatsApp	319	78.8
TV programs	204	50.4
Radio programs	36	8.9
Newspapers	26	6.4
**Frequency of social media use (no. of times/day)**		
≤ 2 times	105	25.9
3–5 times	182	44.9
≥ 6 times	118	29.2
**Duration of social media use (no. of hours/day)**		
≤ 1 h	118	29.1
2–3 h	193	47.7
≥ 4 h	94	23.2

^#^The total does not sum up to 100% due to multiple responses

The relationship between the frequency and duration of social media use and anxiety levels among employees shows a significant increasing trend (*p* < 0.001), indicating that higher social media exposure is associated with greater anxiety severity. Employees who used social media more than six times per day had the highest prevalence of severe anxiety symptoms (27.1% compared to 15.2% among those using it less than two times per day). Similarly, those spending more than 4 h daily on social media had a higher prevalence of severe anxiety symptoms (24.5% compared to 11.9% among those using it for less than 1 h per day). These findings suggest a dose–response relationship, where increased social media exposure is linked to worsening anxiety symptoms (Table 3).

**Table 3:** Relationship between social media use and anxiety levels among studied employees at Ain Shams University (*n* = 405)

Social media use during the day	Anxiety levels								Chi-square for trend (*p*-value)
	**Normal**		**Mild** **anxiety**		**Moderate anxiety**		**Severe** **anxiety**		
	**No.**	**%**	**No.**	**%**	**No.**	**%**	**No.**	**%**	
**Frequency**									
≤ **2 times**	35	33.3	34	32.4	20	19.1	16	15.2	< 0.001*
**3**–**5 times**	26	14.3	72	39.6	55	30.2	29	15.9	
≥ **6 times**	10	8.5	37	31.4	39	33.0	32	27.1	
**Duration**									
≤ **1 h**	40	33.9	43	36.4	21	17.8	14	11.9	< 0.001*
**2**–**3 h**	24	12.4	69	35.8	60	31.1	40	20.7	
≥ **4 h**	7	7.4	31	33.0	33	35.1	23	24.5	

^*^Statistically significant at *p* ≤ 0.05

The relationship between the frequency and duration of social media use and depression levels among employees shows a significant increasing trend (*p* < 0.001) between higher social media exposure and severe depression. Employees who used social media more than six times per day had the highest prevalence of severe depression symptoms (40.7%), compared to 21.0% among those using it less than two times per day). Similarly, employees spending more than 4 h daily on social media had a higher prevalence of severe depression symptoms (41.5%), compared to 16.9% among those using it for less than 1 h per day). These findings suggest a dose–response relationship between increased social media use and worsening depression levels (Table 4).

**Table 4:** Relationship between social media use and depression levels among studied employees at Ain Shams University (*n* = 405)

Social media use during the day	Depression levels								Chi-square for trend (*p*-value)
	**Normal**		**Mild** **depression**		**Moderate depression**		**Severe depression**		
	**No.**	**%**	**No.**	**%**	**No.**	**%**	**No.**	**%**	
**Frequency**									
≤ **2 times**	21	20.0	38	36.2	24	22.8	22	21.0	< 0.001*
**3**–**5 times**	18	9.9	60	33.0	56	30.7	48	26.4	
≥ **6 times**	8	6.8	27	22.9	35	29.6	48	40.7	
**Duration**									
≤ **1 h**	26	22.0	45	38.2	27	22.9	20	16.9	< 0.001*
**2**–**3 h**	15	7.8	53	27.5	66	34.2	59	30.5	
≥ **4 h**	6	6.4	27	28.7	22	23.4	39	41.5	

^*^Statistically significant at *P* ≤ 0.05

The multiple linear regression analysis identifies age, sex, and duration of social media use as significant independent predictors of both Generalized Anxiety Disorder (GAD- 7) and depression (PHQ- 9) scores among employees at Ain Shams University (*p* < 0.001). Employees with higher age had significantly greater anxiety and depression scores compared to younger employees. Similarly, being female was associated with increased anxiety and depression scores. Duration of social media use emerged as the strongest predictor, with longer usage correlating with higher anxiety and depression levels (*β* = 0.460 and *β* = 0.472, respectively). However, the presence of chronic disease did not show a statistically significant association with either outcome (*p* > 0.05). The models demonstrated strong explanatory power, explaining 78.6% of the variance in anxiety scores and 80.8% of the variance in depression scores (*R*^2^ = 0.786 and 0.808, respectively), reinforcing the impact of excessive social media exposure on mental health (Table 5).

**Table 5:** Multiple linear regression displaying independent predictors of generalized anxiety disordersand depression scores among participating employeesat Ain Shams University (*n*** = **405)

**Model**	Unstandardized coefficients		**Standardized coefficients**	***t***	**Sig**
	***B***	Std. error	**Beta**		
**Model 1: Generalized anxiety disorders score (*R***^**2**^** = 0.786, *P* < 0.001)**					
**Age** **(ref. = 20–30 years)**	0.857	0.233	0.222	3.677	< 0.001*
**Sex** **(ref. = male)**	1.510	0.412	0.216	3.663	< 0.001*
**Chronic disease** **(ref. = no)**	0.762	0.621	.041	1.226	0.221
**Duration of social media use** **(ref. = 1 h or less/day)**	2.460	0.285	0.460	8.620	< 0.001*
**Model 2: Depression score (*R***^**2**^** = 0.808, *P* < 0.001)**					
**Age** **(ref. = 20–30 years)**	0.882	0.256	0.197	3.451	< 0.001*
**Sex** **(ref. = male)**	1.907	0.452	0.235	4.221	< 0.001*
**Chronic disease** **(ref. = no)**	1.041	0.681	.048	1.528	0.127
**Duration of social media use** **(ref. = 1 h or less/day)**	2.921	0.313	0.472	9.339	< 0.001*

^*^Statistically significant at *P* ≤ 0.05

## Discussion

The COVID- 19 pandemic posed a serious threat to human life and well-being. Social media served as a primary source of information about COVID- 19. However, it also contributed to the spread of misinformation, increasing fear and anxiety among individuals during the pandemic [18].

The results of the current study revealed that 78.8% of the study participants used social media like Facebook and WhatsApp to get information about the COVID- 19 pandemic. This is in accordance with a similar Egyptian study carried out by Shehata and Abdeldaim (2022) [10] who found that 92.1% of the studied participants obtained the latest pandemic information through social media, especially Facebook. Similar findings were also revealed by a Palestinian study carried out by [11] who discovered that Facebook was used by 81.8% of the survey participants to learn about COVID- 19.

The present study also found that more than half of the participants (56.0%) relied on official websites, while nearly half (50.4%) watched TV programs for information about the COVID- 19 pandemic. In contrast, a German study [6] reported that 79.7% of participants relied on official government and health authority websites for COVID- 19 updates, followed by online news portals (76.9%), highlighting regional differences in information-seeking behavior. This discrepancy may be attributed to the fact that Egyptians tend to use social media more frequently than official websites, as these platforms are more accessible and commonly used for daily communication with family and friends.

Concerning the prevalence of depression and anxiety symptoms among the studied employees, the current study revealed that the majority of study participants 88.4% had depression symptoms, where 30.86% had mild depression and 28.40% and 29.14% had moderate to severe depression respectively as measured by PHQ- 9. Moreover, about 82.47% of the studied participants had anxiety with 35.31% who had mild symptoms, 28.15% had moderate symptoms, and 19.01% had severe symptoms as measured by the GAD- 7 scale.

Comparing these results with another study carried out by Youssef et al., 2020 [19] on 540 health-care professionals working in quarantine and non-quarantine Egyptian hospitals during the COVID- 19 pandemic, it was found that over 50% of the respondents reported some form of adverse psychological symptoms as follows: mild to severe stress 37.2%, depression 59.0%, and anxiety 42.6%.

Another Egyptian study with similar findings [20] among frontline healthcare workers exposed to COVID- 19 revealed that a considerable proportion of healthcare workers had symptoms of anxiety 76.4% as measured by the GAD- 7 scale: 36.2% were mild, 24.4% were moderate, and 15.9% were severe. In addition, 77.2% had depressive symptoms: 35.0% were mild, 23.9% were moderate, and 18.4% were severe.

In contrast, a study in Singapore [21] among “nonmedical” personnel (allied health professionals, pharmacists, technicians, administrators, clerical staff, and maintenance workers) found that lower percentages of anxiety and depression have been reported, where 20.7% of the participants had symptoms of anxiety and 10.3% had symptoms of depression during the COVID- 19 pandemic.

There are several potential reasons why anxiety and depression are more prevalent among administrative workers in developing countries compared to those in developed nations. In developing countries, mental health stigma often discourages individuals from seeking treatment or openly acknowledging their condition due to fear of social judgment and discrimination. As a result, many cases of anxiety and depression remain undiagnosed and untreated, leading to more severe manifestations of these disorders. The lack of early intervention and limited access to adequate mental health care further exacerbate the condition, increasing psychological distress. In contrast, developed countries generally have greater mental health awareness, better healthcare accessibility, and reduced stigma, facilitating early diagnosis and timely treatment, which helps in managing and mitigating the impact of these disorders.

Using social media to stay updated on the pandemic was associated with poorer mental health. This is evident in our study findings, which identified prolonged social media use as an independent predictor of severe depression and severe anxiety. These findings align with those of a German study [6], which reported that individuals relying on social media as their primary information source exhibited significantly higher levels of anxiety and depression. The study also found a significant positive correlation between the daily frequency and duration of media consumption, including social media, and symptoms of anxiety and depression related to COVID- 19. Similarly, a Chinese study [9] demonstrated that frequent social media use was associated with increased odds of anxiety (*OR* = 1.72) and combined depression and anxiety (*OR* = 1.91).

Arabic studies have also reported similar findings. In Egypt, Shehata and Abdeldaim (2022) [10] found that more than 50% of participants believed exposure to pandemic-related news on social media contributed to significant levels of fear and panic. Likewise, a study in Kurdistan, Iraq [12], revealed a strong positive statistical correlation (*r* = 0.87) between social media use and the spread of fear and panic. In Saudi Arabia, Alnohairet al*. (2021)* [22] reported a significant association between pandemic-related media exposure and increased prevalence of depression, anxiety, COVID- 19-related fear, and loneliness. These studies collectively support the current findings, reinforcing the association between frequent social media use and negative mental health outcomes during the pandemic.

### Study limitations

Despite the strengths of this study, several limitations should be acknowledged. First, the cross-sectional design prevents establishing causality between social media use and mental health outcomes, as the direction of the relationship remains unclear. Second, the self-reported nature of social media usage and mental health symptoms may introduce recall bias and social desirability bias, potentially affecting the accuracy of responses. Lastly, although stratified random sampling was employed, the study was limited to administrative personnel at Ain Shams University, which may reduce the generalizability of findings to other occupational groups or the broader population. Future longitudinal studies are recommended to explore causal relationships and assess long-term mental health impacts.

## Conclusions and recommendations

This study highlights a significant association between increased social media use and higher levels of anxiety and depression symptoms among employees at Ain Shams University during the COVID- 19 pandemic. The findings suggest a dose–response relationship, where greater frequency and duration of social media exposure correlate with worsening mental health outcomes. These results emphasize the need to promote responsible social media consumption and raise mental health awareness in workplace settings. Organizations should implement workplace mental health programs, encourage digital well-being strategies, and provide psychological support services to mitigate the adverse effects of excessive social media use.

## Data Availability

Data are available from the corresponding author upon request.

## Abbreviations

COVID- 19: Coronavirus disease 2019
PASS 11.0: Power Analysis and Sample Size Software Version 11.0
PHQ- 9: Patient Health Questionnaire-9
GAD- 7: Generalized Anxiety Disorder 7
IBM SPSS: International Business Machines Statistical Package for the Social Sciences
SD: Standard deviation
*p*-value: Probability value
